# Left and Right Myocardial Functionality Assessed by Two-Dimensional Speckle-Tracking Echocardiography in Cats with Restrictive Cardiomyopathy

**DOI:** 10.3390/ani11061578

**Published:** 2021-05-28

**Authors:** Ryohei Suzuki, Yunosuke Yuchi, Haruka Kanno, Takahiro Teshima, Hirotaka Matsumoto, Hidekazu Koyama

**Affiliations:** Laboratory of Veterinary Internal Medicine, School of Veterinary Medicine, Faculty of Veterinary Science, Nippon Veterinary and Life Science University, Tokyo 180-8602, Japan; y.0301.yunosuke@gmail.com (Y.Y.); cats.haru25pan4@gmail.com (H.K.); teshima63@nvlu.ac.jp (T.T.); matsumoto@nvlu.ac.jp (H.M.); hkoyama@nvlu.ac.jp (H.K.)

**Keywords:** endomyocardial fibrosis, feline, heart, restrictive cardiomyopathy, speckle-tracking echocardiography, strain, strain rate

## Abstract

**Simple Summary:**

The endomyocardial form of restrictive cardiomyopathy, a primary disorder of the myocardium, is one of the diseases with poor prognosis in cats. While two-dimensional speckle-tracking echocardiography has been known to identify myocardial deformations, its function relative to cats with the endomyocardial form of restrictive cardiomyopathy has yet to be characterized. We hypothesized that both the left and right myocardial functional abnormalities may occur in cats with the endomyocardial form of restrictive cardiomyopathy, causing this disease pathophysiology and clinical status. In the current study, cats were assessed for layer-specific myocardial function (whole, endocardial, and epicardial) in the left ventricular longitudinal and circumferential directions, and right ventricular longitudinal direction, via two-dimensional speckle-tracking echocardiography. Our study indicated that cats with restrictive cardiomyopathy have reduced left ventricular myocardial function. Notably, left ventricular systolic circumferential endocardial strain and circumferential endocardial-to-epicardial strain ratio were lower in cats with restrictive cardiomyopathy. Furthermore, some right ventricular myocardial deformations were also differerent in cats with restrictive cardiomyopathy. Myocardial function assessed by two-dimensional speckle-tracking echocardiography could reveal left and right myocardial dysfunction.

**Abstract:**

The endomyocardial form of restrictive cardiomyopathy (EMF-RCM), a primary disorder of the myocardium, is one of the diseases with poor prognosis in cats. We hypothesized that both the left and right myocardial functional abnormalities may occur in cats with EMF-RCM, causing this disease pathophysiology and clinical status. Out of the 25 animals included in this study, 10 were client-owned cats with EMF-RCM, and 15 were healthy cats. In this study, cats were assessed for layer-specific myocardial function (whole, endocardial, and epicardial) in the left ventricular longitudinal and circumferential directions, and right ventricular longitudinal direction, via two-dimensional speckle-tracking echocardiography (2D-STE). Cats with EMF-RCM had depressed left ventricular myocardial deformations both in systole (whole longitudinal strain, epicardial longitudinal strain, and endocardial circumferential strain) and diastole (early and late diastolic longitudinal strain rates, and late diastolic circumferential strain rate) compared to controls. Furthermore, some right ventricular myocardial deformations (systolic longitudinal strain in epicardial layers, and endocardial-to-epicardial strain ratio) were significantly differerent in cats with EMF-RCM. Myocardial function assessed by 2D-STE could reveal left and right myocardial dysfunction.

## 1. Introduction

Restrictive cardiomyopathy is primary cardiomyopathy form in cats, which is classified into the endomyocardial and myocardial forms. The endomyocardial form of restrictive cardiomyopathy (EMF-RCM) is one of the myocardial diseases in cats, characterized by an endomyocardial scar associated with left ventricular (LV) diastolic dysfunction and subsequent left atrial or bi-atrial enlargement [1,2,3]. In human patients with heart disease, myocardial functional assessment is important to distinguish cardiomyopathy from other cause of heart disease [4,5,6]. As feline cardiomyopathy is based on a primary disorder of the myocardium (endomyocardial fibrosis) [1,2,3], myocardial assessment is also important in feline cases for EMF-RCM diagnosis and understanding its pathophysiology. However, detailed assessment of myocardial function in cats with EMF-RCM is yet to be thoroughly recognized.

Recently, we have reported on two-dimensional speckle-tracking echocardiography (2D-STE) applied to cats with hypertrophic cardiomyopathy (HCM) and assessed myocardial functionality in HCM cats [7,8,9]. In these previous studies, the 2D-STE techniques revealed that cats with HCM have myocardial dysfunction, which may reflect characteristics of the HCM myocardium [7,8,9] and layer-specific compensative disease pathophysiology in cats with HCM [7]. We hypothesized that myocardial functional assessment with 2D-STE could reveal more detailed myocardial function, or lack thereof, in cats with EMF-RCM to develop its pathophysiological characteristics.

Many cats with EMF-RCM suffer from congestive heart failure, lethal arrhythmia, and arterial thrombosis, which leads to poor clinical outcomes [10,11]. While recent studies suggested that right heart involvement with disease pathophysiology contributed to severe clinical status in cats with HCM [12,13], right heart function in cats with EMF-RCM has not yet been evaluated. We hypothesized that both left and right myocardial functional abnormalities may contribute to the pathophysiology of EMF-RCM in cats, resulting in its relevant clinical status. Therefore, the aim of this study was to evaluate left and right myocardial function using 2D-STE in cats with EMF-RCM.

## 2. Materials and Methods

### 2.1. Animals

We prospectively included cats clinically diagnosed with EMF-RCM and healthy cats serving as controls during the period between November 2018 and September 2020. Cats with asymptomatic and symptomatic but stable conditions were allowed in this study. All cats underwent complete physical examination, electrocardiography, thoracic radiography, blood pressure measurement, and trans-thoracic echocardiography. The diagnostic criteria of EMF-RCM were the presence of (1) echocardiographic evidence of left atrial or bi-atrial enlargement and (2) prominent endomyocardial scar that bridges the interventricular septum and LV free wall. Left atrial enlargement was defined by a left atrial-to-aortic-root ratio (LA/Ao) greater than 1.5, from the right parasternal short-axis view using the B-mode method [14]. Right atrial enlargement was judged on the right parasternal long-axis view according to previously published allometric scaling reference intervals [12]. Endomyocardial scar finding was macroscopically judged by B-mode echocardiography. To exclude other forms of feline cardiomyopathy [1], we checked normal or near-normal LV systolic function and wall thickness according to previously published allometric scaling reference intervals [15]. Therefore, cats with obvious systolic dysfunction or LV focal and diffuse hypertrophy were not allowed in this study. Furthermore, while cats with systolic anterior motion of the mitral valve, persistent arrhythmias, congenital heart disease, hyperthyroidism, and systemic hypertension (noninvasive systolic blood pressure >160 mmHg) were excluded from the study, those receiving cardiac medications were allowed.

Healthy cats were identified by normal findings pertaining to the aforementioned examinations. None of the healthy cats were on medication or had a history of clinical signs of heart disease. All procedures in this study followed the Guidelines for University Hospital Animal Care of Nippon Veterinary and Life Science University in Japan, and were approved by the ethical committee of our institute (approval number: R2–4). All owners of recruited cats provided consent for research use of their clinical data.

### 2.2. Standard Echocardiography

Standard 2D and Doppler examinations were performed by a single trained investigator (R.S.) using a Vivid E95 echocardiographic system (GE Healthcare, Tokyo, Japan) and a 3.5–6.9 MHz transducer (GE Healthcare, Tokyo, Japan); the Lead II ECG was recorded simultaneously and displayed on the images. All echocardiographic data were obtained from at least five consecutive cardiac cycles in sinus rhythm from non-sedated cats that were manually restrained in the right and left lateral recumbent positions. Diagnosis of EMF-RCM was made during the examination with the agreement by two independent cardiologists (R.S. and H.Ko.). For this research, the echocardiographic images were analyzed, on a separate day from the examination, by a single trained observer (H.Ka.), who remained unaware of the examination data of the cats, using an offline workstation (EchoPAC PC, Version 201, GE Healthcare, Tokyo, Japan). End-diastolic interventricular septal thickness, end-diastolic LV free-wall thickness, end-diastolic LV internal diameter, end-systolic LV internal diameter, and fractional shortening were measured using the B-mode method from the right parasternal short-axis view of the LV. Trans-mitral inflow was obtained by the pulsed wave Doppler method from the left apical 4-chamber view, and the peak velocity of the early diastolic wave (E-wave), and peak velocity of the late diastolic wave (A-wave) were measured. Values were not used from cats whose E and A waves were fused. End-diastolic right ventricular (RV) internal dimension, end-diastolic RV free wall thickness, and tricuspid annulus plane systolic excursion were measured by B-mode method from the left apical 4-chamber view modified for right heart measurement [12,13]. RV fractional area change was also measured by directly traced end-diastolic and end-systolic RV internal area from the left apical 4-chamber view modified for right heart measurement [13]. Acceleration-time-to-ejection-time ratio of the pulmonary artery was also calculated from the right parasternal short-axis view of the LV [16]. Systolic and early diastolic myocardial velocity of septal mitral annulus and tricuspid annulus was obtained by pulsed wave based on tissue Doppler method from the left apical 4-chamber view and the left apical 4-chamber view modified for right heart measurement [17]. Trans-mitral E-wave velocity to early diastolic myocardial velocity of septal mitral annulus ratio was also evaluated. For all analyses, the mean values of three consecutive cardiac cycles from high-quality images were used.

### 2.3. Two-Dimensional Speckle-Tracking Echocardiography

**Figure 1:**
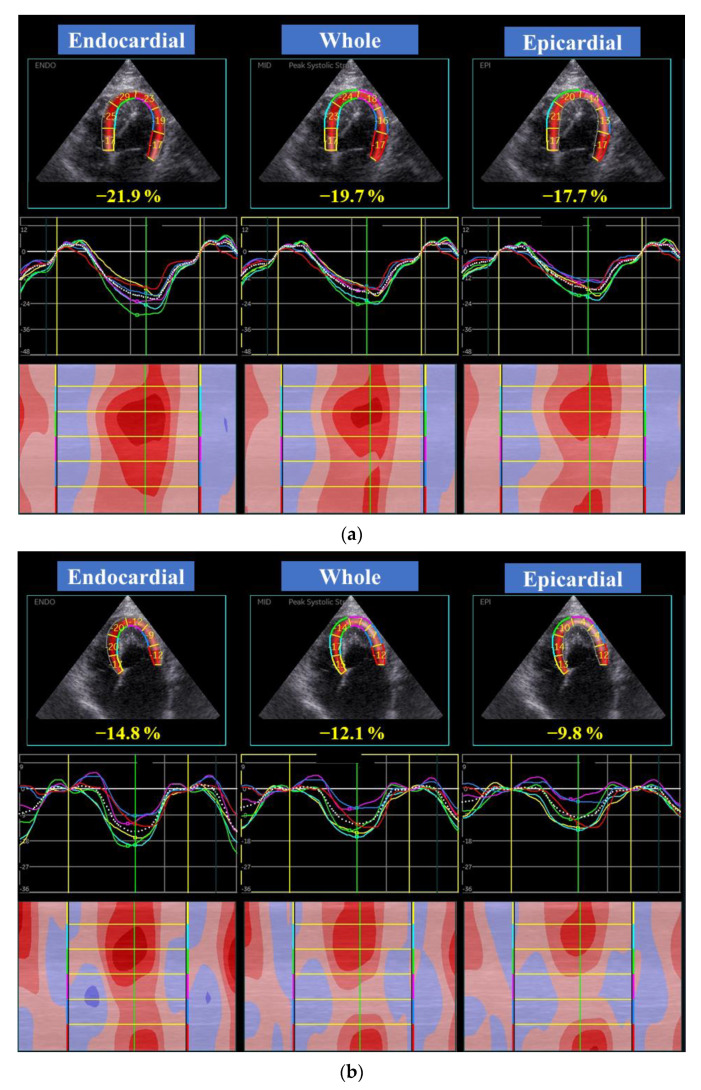
Layer-specific (whole, endocardial, and epicardial layer) left ventricular longitudinal global (dotted line) and segmental (colored lines) strain curves obtained from two-dimensional speckle tracking echocardiography (left apical four-chamber) in a healthy cat (**a**) and a cat with the endomyocardial form of restrictive cardiomyopathy (**b**). Six segmental curves are designated as the basal septum (yellow), middle septum (light blue), apical septum (green), apical lateral (purple), middle lateral (dark blue), and basal lateral (red) for speckle tracking analysis.

**Figure 2:**
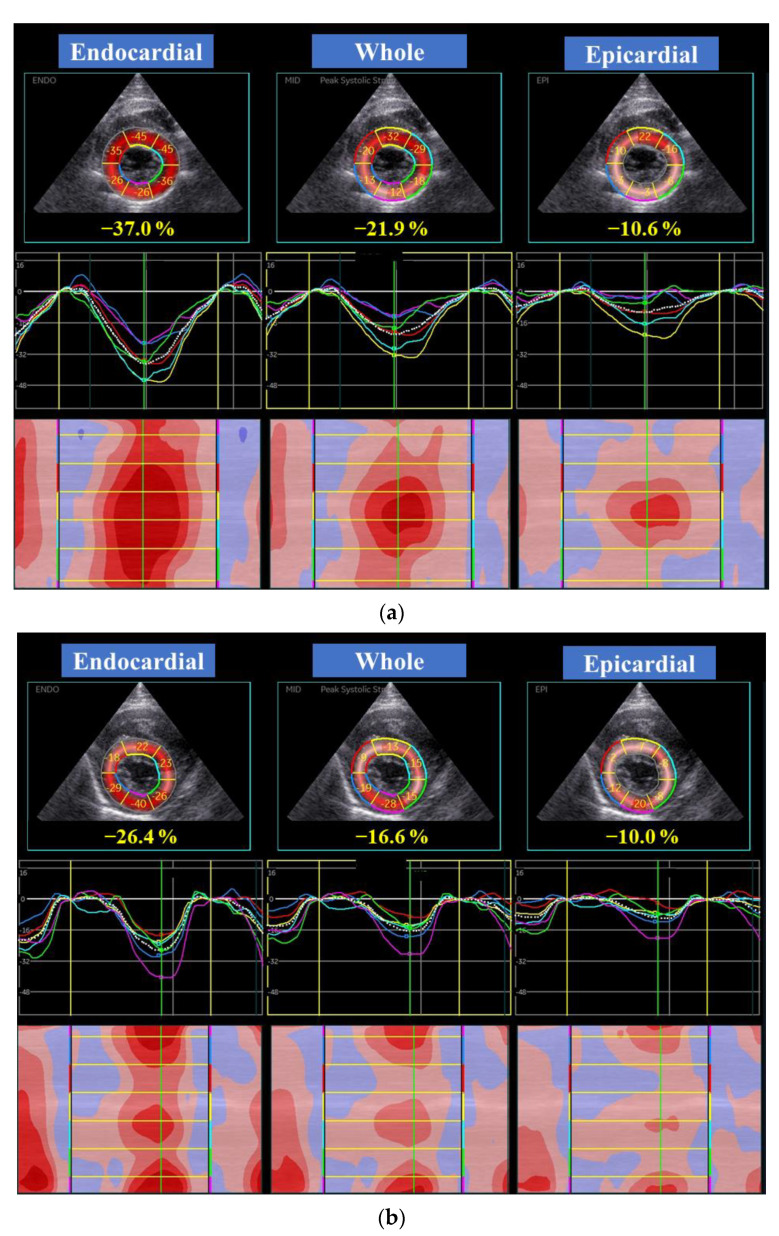
Layer-specific (whole, endocardial, and epicardial layer) left ventricular circumferential global (dotted line) and segmental (colored lines) strain curves obtained from two-dimensional speckle tracking echocardiography (right parasternal short-axis view) in a healthy cat (**a**) and a cat with the endomyocardial form of restrictive cardiomyopathy (**b**). Six segmental curves are designated as the anterior septum (yellow), anterior (light blue), lateral (green), posterior (purple), inferior (dark blue), and septum (red) for speckle tracking analysis. Note the depressed endocardial circumferential strain in this representative endomyocardial form of restrictive cardiomyopathy cat.

**Figure 3:**
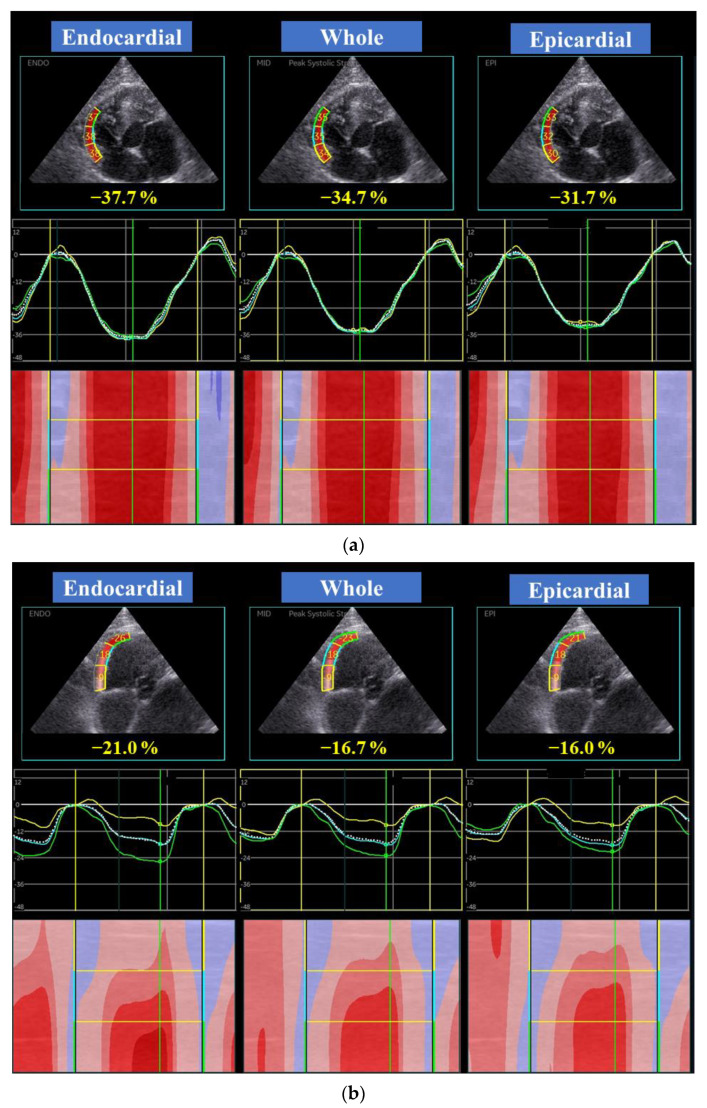
Layer-specific (whole, endocardial, and epicardial layer) right ventricular free wall longitudinal global (dotted line) and segmental (colored lines) strain curves obtained from two-dimensional speckle tracking echocardiography (left apical four-chamber view modified for right heart assessment) in a healthy cat (**a**) and a cat with the endomyocardial form of restrictive cardiomyopathy (**b**). Three segmental curves are designated as the basal lateral (yellow), middle lateral (light blue), apical lateral (green) in the right ventricular free wall for speckle tracking analysis. Note that all layers are depressed in right ventricular strains in this representative endomyocardial form of restrictive cardiomyopathy in cats.

High-quality images for 2D-STE analysis were carefully obtained by the same investigator using the same echocardiographic system and same transducer. To evaluate LV myocardial deformations, a right parasternal short-axis view of the LV at the level of the papillary muscles and a left apical 4-chamber view were obtained in this study (Figure 1 and Figure 2). A left apical 4-chamber view modified for right heart measurement was also obtained to analyze right myocardial deformations (Figure 3). All 2D-STE analyses were performed on a separate day from the examination by a single trained observer (H.Ka.) who was blinded to the examination data of the cats, using an offline EchoPAC workstation. The outline of feline 2D-STE analysis was described previously [7,8,9,18,19]. We measured the peak systolic LV strain of the endocardial, whole, and epicardial layers in the longitudinal and circumferential directions. We calculated the LV endocardial-to-epicardial strain ratio to determine the endocardial dependency of the myocardium [20,21]. We also measured the peak systolic strain rate as well as both early and late diastolic strain rates in the longitudinal and circumferential directions. For RV deformations, we assessed RV lateral wall segments only, and measured peak systolic strain and peak systolic strain rate, as well as both early and late diastolic strain rates in the longitudinal direction. The mean values of the measurements from three consecutive cardiac cycles from high-quality images were used in all analyses.

### 2.4. Reproducibility of 2D-STE Variables

Intra-observer measurement of variability was performed by the same observer who performed all echocardiographic analyses. All of the 2D-STE indices from three healthy cats and three EMF-RCM cats, selected at random, were measured on different days. The studies were also analyzed by a second blinded observer (Y.Y.) to assess inter-observer reproducibility.

### 2.5. Statistical Analysis

Data were reported as medians and interquartile ranges. Statistical analyses were performed using R software version 2.8.1. (The R Foundation for Statistical Computing, Vienna, Austria). The normality of data was tested using the Shapiro–Wilk test. Chi-squared or Fisher’s exact tests were used to compare categorical indices between the groups. We used a Student’s T-test or Mann–Whitney U-test to compare the variables between EMF-RCM and control cats. Intra- and inter-observer measurement variability was quantified by the coefficient of variation. Reproducibility was also evaluated by intra- and inter-class correlation coefficients for intra- and inter-observer measurement variability. Statistical significance was set at *p* < 0.05.

To evaluate the validity of the sample size, a power analysis was performed as a post hoc analysis in echocardiographic indices which showed significant differences between each group. The power was evaluated using mean difference between two groups, standard deviation, and sample size. An α error was set at 0.05. Sufficient power to detect the difference was set at >0.80.

## 3. Results

### 3.1. Clinical Profiles and Standard Echocardiography

We diagnosed 13 cats with an EMF-RCM classification of cardiomyopathy during the study period, but two cats had poor echocardiographic images and one cat had atrial fibrillation, thus these were excluded. Finally, 10 cats with EMF-RCM (stage B2: *n*= 2, stage C: *n* = 8, [1]) and 15 healthy cats serving as controls were enrolled in this study. Staging was according to American College of Veterinary Internal Medicine consensus: Stage B2 identified asymptomatic cats with significant left atrial enlargement based on echocardiography, and Stage C identified symptomatic cats with current or past clinical findings of heart failure caused by cardiomyopathy. The breeds of cats with EMF-RCM were domestic shorthair (*n* = 7), Maine coon (*n* = 1), Himalayan (*n* = 1), and Singapura (*n* = 1). One cat with EMF-RCM had past evidence of cardiac heart failure (pulmonary edema). Seventy percent of the cats with EMF-RCM had current evidence of cardiac heart failure (mild pulmonary edema (*n* = 2), mild pleural effusion (*n* = 1)), both pulmonary edema and pleural effusion (*n* = 3), or atrial thrombosis (*n* = 1)), but respiratory distress symptoms were controlled using follow-up oral medications. Seventy percent of cats with EMF-RCM were receiving medical treatment from the referral hospital at the time of examination (loop diuretics (*n* = 4), pimobendan (*n* = 4), angiotensin-converting enzyme inhibitors (*n* = 4), prostacyclin derivative formulations (*n* = 1), beta-blocker (*n* = 1), and antithrombotic drugs (*n* = 4)). In the EMF-RCM group, four had arrhythmia, measured via ECG, at the time of examination, including non-sustained ventricular premature complex (*n* = 3) and non-sustained ventricular tachycardia (*n* = 1). Echocardiographic images in cats with non-sustained ventricular premature complex or ventricular tachycardia were assessed in sinus rhythm. EMF-RCM cats had echocardiographic abnormal findings, including spontaneous echo contrast (*n* = 7), mitral regurgitation (*n* = 3), tricuspid regurgitation (*n* = 2), and mitral and tricuspid regurgitation (*n* = 3). We judged pulmonary hypertension had occurred in two cats based on tricuspid regurgitation velocities of more than 2.7 m/s [16].

The breeds within the healthy group of felines consisted of the domestic shorthair (*n* = 7), Main Coon (*n* = 2), Himalayan (*n* = 2), American shorthair (*n* = 1), Russian blue (*n* = 1), sphinx (*n* = 1) and Scottish fold (*n* = 1). None of the control cats were on medications or had past or current signs of congestive heart failure, arrhythmia, intracardiac thrombosis, or spontaneous echo contrast findings.

The clinical profiles and standard echocardiographic data in healthy cats and cats with EMF-RCM are shown in Table 1. Age, body weight, heart rate, and systolic blood pressure did not differ significantly between EMF-RCM and healthy cats. The EMF-RCM cats had significantly higher measurements of LA/Ao, end-diastolic LV internal diameter, trans-mitral E-wave velocity, E/A ratio, and trans-mitral E-wave to early-diastolic myocardial velocity of mitral annulus ratio. They also had significantly higher measurements of right atrial diameter, end-diastolic RV internal dimension, and end-diastolic RV free wall thickness. In contrast, fractional shortening, systolic myocardial velocity of the mitral annulus, and trans-mitral A-wave velocity were significantly lower in cats with EMF-RCM than in controls. Also, they had significantly lower systolic myocardial velocity of the tricuspid annulus, tricuspid annulus plane systolic excursion, and acceleration-time-to-ejection-time ratio of the pulmonary artery, but did not differ for RV fractional area change.

### 3.2. Two-Dimensional Speckle-Tracking Echocardiography

All views for the analysis of 2D-STE were recorded at an average rate of 157 (range, 134 to 170) frames/s, which is adequate for speckle-tracking evaluation of cats [7,8,9,22]. The average coefficients of variation and intra- and inter-class correlation coefficient for intra- and inter-observer reliability for 2D-STE assessment are summarized in Table 2.

**Figure 4:**
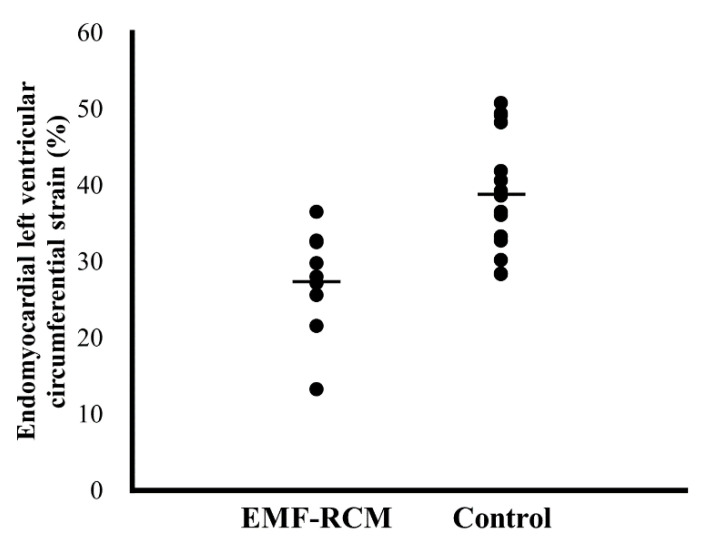
Individual plots of left ventricular circumferential strain in endomyocardial layer in cats with restrictive cardiomyopathy and healthy cats. Bar represents mean values of each group. EMF-RCM, endomyocardial form of restrictive cardiomyopathy.

The systolic 2D-STE data for healthy cats and those with EMF-RCM are shown in Table 3. Global LV longitudinal strain was significantly lower in the EMF-RCM group than in controls, in whole and epicardial layers. The global LV circumferential strain in the endocardial layer and endocardial-to-epicardial strain ratio also were significantly lower in the EMF-RCM group than in the control group. Individual plots of LV circumferential strain in endomyocardial layer in cats with EMF-RCM and healthy cats are shown in Figure 4. Furthermore, the EMF-RCM group had significantly lower epicardial longitudinal strain, and higher endocardial-to-epicardial strain ratio. All echocardiographic variables which showed statistical significance except for RV epicardial strain had sufficient powers to detect the differences.

The diastolic 2D-STE data for healthy cats and cats with EMF-RCM are shown in Table 4. Early and late diastolic LV longitudinal strain rates were significantly lower in the EMF-RCM group than in the control group. The EMF-RCM group also had significantly lower late diastolic LV circumferential strain rate. All echocardiographic variables which showed statistical significance except for the LV longitudinal strain rate in late diastole had sufficient powers to detect the differences.

## 4. Discussion

We found that 2D-STE myocardial assessment could be applied to cats with EMF-RCM with adequate repeatability. Our study indicated that cats with EMF-RCM have depressed LV myocardial deformations both in systole and diastole. Notably, LV systolic circumferential endocardial strain and subsequent circumferential endocardial-to-epicardial strain ratio were lower in cats with EMF-RCM, which may reflect the pathophysiological changes of endomyocardial fibrosis in the EMF-RCM myocardium. Furthermore, some RV myocardial deformations were also differerent in cats with EMF-RCM.

In our study, global LV myocardial deformations were significantly lower in cats with EMF-RCM compared to the control group both in systole and diastole. Although myocardial function in cats with EMF-RCM had not been reported to date, this myocardial dysfunction may have been altered by the restrictive characteristics of the EMF-RCM myocardium. Previous studies in humans indicated that low myocardial deformations assessed via 2D-STE were a good indication for distinguishing RCM from constrictive pericarditis [4,5,6]. Likely, decreased systolic and diastolic myocardial deformations may be characteristic findings in cats with EMF-RCM, making the 2D-STE yet another useful assessment for RCM diagnosis. In human patients with cardiomyopathy, deterioration of myocardial strain was identified as an independent predictor of adverse outcomes [23,24]. In our study, EMF-RCM cats already presented depressed myocardial function and most cats had suffered from cardiac heart failure signs. Although clinical outcome was not assessed in our study, severe myocardial dysfunction may, nonetheless, lead to poor clinical outcome in cats with RCM. The clinical importance of myocardial deformations as assessed by 2D-STE and their relationship to survival time should be investigated. In addition, from a clinical point of view, the detection of asymptomatic cats with RCM is required. Future study that includes a greater number of asymptomatic cats with RCM is warranted to identify the usefulness of the early detection.

Circumferential deformations play an important role in cardiac pump function in humans and dogs with cardiac disease [25,26,27], and LV myocardial contractions that are impaired in the longitudinal direction are compensated for by circumferential shortening in subclinical patients with cardiovascular risk factors [28]. The endocardial-to-epicardial circumferential strain ratio may reflect endocardial dependency for compensation of the myocardium [20,21]. Previous feline studies suggested that endocardial circumferential strain tended to be passively maintained by the epicardial layer [7,18]. Further, the endocardial-to-epicardial circumferential strain ratio is high in cats with compensative asymptomatic HCM, owing to the layer-to-layer compensatory mechanism [7,18]. In the present study, layer-specific assessment revealed that LV systolic endocardial circumferential strain and subsequent endocardial-to-epicardial circumferential strain ratio were lower in cats with EMF-RCM. Thus, endomyocardial circumferential dysfunction may be caused by the pathophysiological myocardial changes of EMF-RCM (i.e., endocardial myocardial fibrosis). In contrast to a previous study about HCM, in which higher endomyocardial function was typical in cats with HCM disease [7], depressed endomyocardial circumferential function was typical in cats with EMF-RCM. These myocardial functional abnormalities found in this study may provide additional tools to distinguish the type of feline cardiomyopathy.

Epicardial RV longitudinal myocardial strain and endocardial-to-epicardial strain ratio were differerent in cats with EMF-RCM. These results suggest that the EMF-RCM could be caused by myocardial fibrosis as with LV. Furthermore, RV myocardium could have been affected by pulmonary hypertension secondary to left ventricular filling disorder [16], although there were only two cats diagnosed with pulmonary hypertension based on tricuspid regurgitation >2.7 m/s. As the Doppler-derived detection of pulmonary hypertension complications in cats with cardiomyopathy is difficult in clinical settings [16], right myocardial assessment using 2D-STE may be useful to detect occult complications of pulmonary hypertension in cats. However, the results of right ventricular strains may be affected by the number of included cats. Future research that includes a greater number of cats with RCM is expected to further validate our findings.

This study has several limitations. First, because it was a noninvasive clinical investigation, we did not have the histopathological findings to make a definitive diagnosis and assess myocardial histopathological alterations. Second, we could not consider the influences of medication on the values assessed by 2D-STE because our hospital provided secondary medical care, and as a result, many cats had received some drugs prior to examination. These medications could affect myocardial performance, thereby complicating the interpretation of our results. In particular, pimobendan and beta-blocker agents may directly affect the myocardial functionality. Third, the small number of cats in our study may have had an influence on statistical power and limited extrapolation of our findings to larger populations. However, we obtained detailed and precise myocardial functional data in cats with EMF-RCM in our study. Furthermore, especially left myocardial abnormalities which showed significant differences had sufficient power to detect the differences and the number of cats included was verified. Fourth, RCM cats also had various pathophysiology other than cardiomyopathy, such as heart failure, lung edema, and pulmonary hypertension. These confounding factors made it difficult to interpret our results. Finally, we have evaluated only longitudinal RV strain and strain rate. RV circumferential function would also contribute to RV systolic function in addition to the longitudinal function [29,30]. Further studies are needed to assess this component of RV function in cats affected by RCM. These limitations should be overcome in future investigations.

## 5. Conclusions

Myocardial functionality assessed by the novel 2D-STE technique could reveal left and right myocardial dysfunction in feline patients with EMF-RCM. These myocardial functional abnormalities may be caused by pathophysiological alterations in cats with EMF-RCM. Decreased systolic and diastolic myocardial deformations in both the left and right myocardium may be characteristic findings in cats with EMF-RCM and may also be useful in its diagnosis. Principally, depressed endomyocardial circumferential function is typical in cats with EMF-RCM, unlike previous results in cats with HCM. Overall, measurement of myocardial functional assessment using 2D-STE may provide more detailed and novel findings for cats with EMF-RCM. Nevertheless, the clinical importance of these myocardial functional abnormalities and their relationship to survival time need further research.

## Figures and Tables

**Table 1:** Clinical profiles and standard echocardiographic data in healthy cats and cats with the endomyocardial form of restrictive cardiomyopathy.

	EMF-RCM		*n*	Control		*n*	*p*
Age (month)	82.5	(41.5, 164.3)	10	69.0	(25.0, 140.0)	15	0.08
Body weight (kg)	4.7	(4.0, 5.2)	10	4.2	(3.5, 4.5)	15	0.16
Heart rate (bpm)	218	(167, 240)	10	203	(181, 225)	15	0.96
Systolic blood pressure (mmHg)	145	(121, 160)	10	140	(125, 149)	15	0.66
Gender (male/female)	6/4			8/7			1.00
LA/Ao	2.6	(2.1, 3.2)	10	1.3	(1.2, 1.4)	15	<0.01
IVSd (mm)	4.0	(2.7, 5.1)	10	3.8	(3.4, 4.1)	15	0.71
LVIDd (mm)	16.8	(15.1, 17.6)	10	14.0	(13.5, 15.5)	15	<0.01
LVFWd (mm)	4.1	(3.5, 5.1)	10	4.2	(3.6, 4.3)	15	0.80
FS (%)	34.0	(26.4, 40.3)	10	49.1	(40.8, 53.1)	15	<0.01
E-wave velocity (cm/s)	0.82	(0.79, 0.85)	9	0.60	(0.57, 0.73)	12	<0.01
A-wave velocity (m/s)	0.30	(0.24, 0.39)	9	0.69	(0.57, 0.73)	12	<0.01
E/A ratio	2.9	(2.5, 3.4)	9	0.9	(0.8, 1.2)	12	<0.01
MV s’ (cm/s)	5.2	(4.6, 5.5)	8	8.0	(6.9, 10.5)	12	0.02
MV e’ (cm/s)	5.8	(4.9, 6.1)	8	7.0	(5.1, 7.6)	12	0.10
MV E/e’	14.1	(13.0, 16.2)	8	9.4	(8.2, 12.3)	12	<0.01
Right atrial diameter (mm)	11.2	(10.1, 13.9)	10	9.1	(8.3, 10.7)	15	0.02
RVIDd (mm)	8.8	(7.8, 9.1)	10	7.1	(6.2, 7.6)	15	0.047
RVFWd (mm)	2.3	(2.2, 2.7)	10	1.9	(1.7, 2.1)	15	<0.01
TAPSE (mm)	6.1	(5.0, 8.4)	10	9.1	(7.6, 10.9)	15	<0.01
RV FAC (%)	53.3	(39.2, 60.0)	10	57.0	(51.6, 63.0)	15	0.12
RV s’ (cm/s)	7.4	(6.4, 8.8)	10	10.5	(8.4, 12.2)	14	0.07
RV e’ (cm/s)	10.7	(7.1, 15.7)	10	9.4	(6.7, 11.0)	14	0.64
PV AT/ET	0.33	(0.22, 0.45)	10	0.50	(0.43, 0.53)	15	0.01

Data are shown as medians (25%, 75% interquartile ranges). EMF-RCM, the endomyocardial form of restrictive cardiomyopathy; LA/Ao, left-atrial-to-aortic-root ratio; IVSd, end-diastolic interventricular septal thickness; LVIDd, end-diastolic left ventricular internal diameter; LVFWd, end-diastolic left ventricular free wall thickness; FS, fractional shortening; MV s’, systolic myocardial velocity of septal mitral annulus; MV e’, early-diastolic myocardial velocity of septal mitral annulus; MV E/e’, trans-mitral E-wave velocity to early-diastolic myocardial velocity of septal mitral annulus ratio; RVIDd, end-diastolic right ventricular internal dimension; RVFWd, end-diastolic right ventricular free wall thickness; TAPSE, tricuspid annulus plane systolic excursion; RV FAC, right ventricular fractional area change; RV s’, systolic myocardial velocity of tricuspid annulus; RV e’, early-diastolic myocardial velocity of tricuspid annulus; and PV AT/ET, acceleration-time-to-ejection-time ratio of the pulmonary artery.

**Table 2:** Results of intra- and inter-observer reliability for two-dimensional speckle-tracking echocardiography assessment.

	Intra-Observer		Inter-Observer	
	CV (%)	ICC	CV (%)	ICC
Global LV systolic longitudinal strain				
Whole layer	2.2	0.98 *	1.8	0.98 *
Endocardial layer	1.3	0.99 *	3.1	0.95 *
Epicardial layer	2.6	0.97 *	3.6	0.94 *
Global LV systolic longitudinal strain rate	4.0	0.94 *	5.4	0.90 *
Global LV early-diastolic longitudinal strain rate	7.5	0.91 *	5.1	0.96 *
Global LV late-diastolic longitudinal strain rate	7.4	0.99 *	14.4	0.97 *
Global LV systolic circumferential strain				
Whole layer	4.6	0.92 *	6.6	0.91 *
Endocardial layer	6.5	0.95 *	5.8	0.96 *
Epicardial layer	9.2	−0.16	10.7	0.03
Global LV systolic circumferential strain rate	5.2	0.85 *	5.7	0.84 *
Global LV early-diastolic circumferential strain rate	7.8	0.38	10.5	−0.41
Global LV late-diastolic circumferential strain rate	23.9	0.38	9.4	0.97 *
Global RV systolic longitudinal strain				
Whole layer	3.7	0.98 *	5.4	0.98 *
Endocardial layer	3.3	0.99 *	5.1	0.98 *
Epicardial layer	3.8	0.98 *	5.8	0.97 *
Global RV systolic longitudinal strain rate	16.4	0.40	12.3	0.80
Global RV early-diastolic longitudinal strain rate	13.4	0.58	13.5	0.78 *
Global RV late-diastolic longitudinal strain rate	20.3	0.92 *	9.2	0.95 *

* Within a row, ICC values were considered significant (*p* < 0.05). CV, coefficient of variation; ICC, intra- or inter-class correlation coefficients; LV, left ventricular; RV, right ventricular.

**Table 3:** The systolic two-dimensional speckle-tracking echocardiography data for healthy cats and cats with the endomyocardial form of restrictive cardiomyopathy.

	EMF-RCM		*n*	Control		*n*	*p*
Global LV longitudinal strain (%)							
Whole layer	13.4	(11.4, 20.8)	10	20.8	(18.3, 22.6)	15	0.04
Endocardial layer	16.3	(13.6, 23.6)	10	23.8	(21.9, 25.3)	15	0.08
Epicardial layer	11.8	(9.2, 17.3)	10	18.5	(16.1, 19.8)	15	<0.01
Endo/Epi	1.43	(1.34, 1.55)	10	1.36	(1.2, 1.4)	15	0.03
Global LV longitudinal strain rate (%/s)	2.8	(1.8, 3.0)	10	2.9	(2.3, 3.5)	15	0.10
Global LV circumferential strain (%)							
Whole layer	17.4	(15.4, 21.3)	10	19.1	(17.3, 23.9)	15	0.09
Endocardial layer	27.7	(25.4, 32.6)	10	38.7	(32.7, 48.3)	15	<0.01
Epicardial layer	10.4	(8.5, 12.3)	10	7.3	(6.2, 9.3)	15	0.14
Endo/Epi	2.73	(2.08, 4.06)	10	4.77	(4.12, 6.50)	15	<0.01
Global LV circumferential strain rate (%/s)	2.7	(2.2, 3.0)	10	2.8	(2.5, 3.2)	15	0.14
Global RV longitudinal strain (%)							
Whole layer	22.9	(16.4, 31.9)	10	31.6	(27.8, 39.1)	15	0.07
Endocardial layer	24.9	(19.0, 34.2)	10	34.8	(29.6, 42.3)	15	0.10
Epicardial layer	21.3	(14.0, 29.8)	10	29.0	(26.0, 36.4)	15	0.045
Endo/Epi	1.21	(1.13, 1.30)	10	1.15	(1.13, 1.18)	15	0.03
Global RV longitudinal strain rate (%/s)	4.0	(3.7, 5.5)	10	5.5	(4.8, 6.7)	15	0.17

Data are shown as medians (25%, 75% interquartile ranges). EMF-RCM, endomyocardial form of restrictive cardiomyopathy; LV, left ventricular; RV, right ventricular; Endo/Epi, endocardial-to-epicardial strain ratio.

**Table 4:** The diastolic two-dimensional speckle-tracking echocardiography data for healthy cats and cats with the endomyocardial form of restrictive cardiomyopathy.

	EMF-RCM		*n*	Control		*n*	*p*
Early-diastolic LV longitudinal strain rate (%/s)	4.1	(2.6, 4.3)	9	5.2	(4.1, 5.8)	8	<0.01
Late-diastolic LV longitudinal strain rate (%/s)	1.3	(0.3, 2.1)	9	2.3	(1.5, 4.1)	8	0.049
Early-diastolic LV circumferential strain rate (%/s)	3.7	(2.8, 4.7)	8	3.4	(3.0, 4.0)	15	0.45
Late-diastolic LV circumferential strain rate (%/s)	0.9	(0.2, 1.9)	8	2.7	(1.7, 3.4)	15	<0.01
Early-diastolic RV longitudinal strain rate (%/s)	5.5	(4.4, 6.3)	10	5.6	(4.8, 6.7)	13	0.29
Late-diastolic RV longitudinal strain rate (%/s)	1.9	(1.1, 5.2)	10	4.6	(3.8, 6.6)	13	0.11

Data are shown as medians (25%, 75% interquartile ranges). EMF-RCM, endomyocardial form of restrictive cardiomyopathy; LV, left ventricular; RV, right ventricular.
